# Evolutionary Dynamics of the Interferon-Induced Transmembrane Gene Family in Vertebrates

**DOI:** 10.1371/journal.pone.0049265

**Published:** 2012-11-15

**Authors:** Zhao Zhang, Jun Liu, Meng Li, Hui Yang, Chiyu Zhang

**Affiliations:** 1 Institute of Life Sciences, Jiangsu University, Jiangsu, China; 2 State Key Laboratory of Genetic Resources and Evolution, Kunming Institute of Zoology, Chinese Academy of Sciences, Kunming, China; 3 Diagnostic center for pathogens, Institut Pasteur of Shanghai, Shanghai Institutes for Biological Sciences, Chinese Academy of Sciences, Shanghai, China; University of Lausanne, Switzerland

## Abstract

Vertebrate interferon-induced transmembrane (IFITM) genes have been demonstrated to have extensive and diverse functions, playing important roles in the evolution of vertebrates. Despite observance of their functionality, the evolutionary dynamics of this gene family are complex and currently unknown. Here, we performed detailed evolutionary analyses to unravel the evolutionary history of the vertebrate IFITM family. A total of 174 IFITM orthologous genes and 112 pseudogenes were identified from 27 vertebrate genome sequences. The vertebrate IFITM family can be divided into immunity-related IFITM (IR-IFITM), IFITM5 and IFITM10 sub-families in phylogeny, implying origins from three different progenitors. In general, vertebrate IFITM genes are located in two loci, one containing the IFITM10 gene, and the other locus containing IFITM5 and various numbers of IR-IFITM genes. Conservation of evolutionary synteny was observed in these IFITM genes. Significant functional divergence was detected among the three IFITM sub-families. No gene duplication or positive selection was found in IFITM5 sub-family, implying the functional conservation of IFITM5 in vertebrate evolution, which is involved in bone formation. No IFITM5 locus was identified in the marmoset genome, suggesting a potential association with the tiny size of this monkey. The IFITM10 sub-family was divided into two groups: aquatic and terrestrial types. Functional divergence was detected between the two groups, and five IFITM10-like genes from frog were dispersed into the two groups. Both gene duplication and positive selection were observed in aquatic vertebrate IFITM10-like genes, indicating that IFITM10 might be associated with the adaptation to aquatic environments. A large number of lineage- and species-specific gene duplications were observed in IR-IFITM sub-family and positive selection was detected in IR-IFITM of primates and rodents. Because primates have experienced a long history of viral infection, such rapid expansion and positive selection suggests that the evolution of primate IR-IFITM genes is associated with broad-spectrum antiviral activity.

## Introduction

First discovered by cDNA library screening in 1984 [1], the interferon-induced transmembrane (IFITM) gene family plays critical roles in a variety of cellular processes and contains IFITM1, IFITM2, IFITM3, IFITM5, IFITM6, IFITM7, IFITM10 and some IFITM-like genes [2]. Except for IFITM5 that is specifically expressed in bone cells in an interferon (IFN)-independent way [3], [4], all IFITM genes can be stimulated by IFN [5], [6], and are widely expressed in tissues and organs [2].

**Figure 1:**
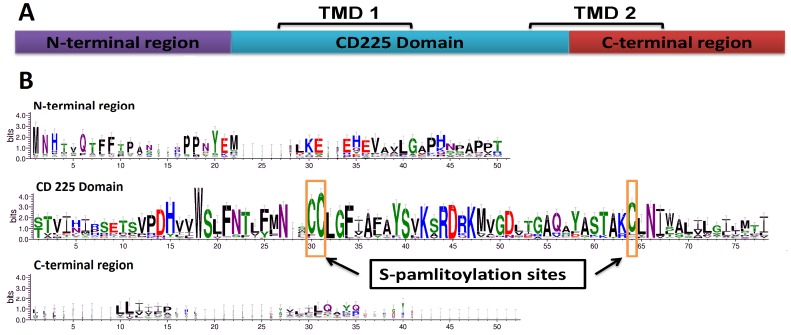
Domain analysis and sequence characteristics of IFITM gene family. Sequence logos were generated based on the alignment of all IFITM and IFITM-like genes identified (160 sequences) in this study. (**A**) Gene structure of all IFITM and IFITM-like genes. (**B**) Logos of the C-terminal region, CD225 domain and N-terminal region.

IFITM family members contain a conservative CD225 domain and two terminal hypervariable regions [2]. The CD225 domain accounts for more than half of the protein in length, containing one intact transmembrane domain (TMD), two S-palmitoylation sites regions and partial TMD in the C-terminus of the protein. The S-palmitoylation sites have been demonstrated to play important roles in post-translational processing and stability of IFITM proteins [7]. The N-terminal hypervariable region generally contains 21 amino acid residues and the C-terminal one includes a TMD (Fig. 1) [8].

In different vertebrates, the functions of different IFITM members diverge. IFITM1, IFITM2 and IFITM3 are involved in cell adhesion [9], antiproliferation [9], tumor suppression [10], [11], and germ cell and embryonic development [12]. More recently, these genes were identified as novel types of antiviral restriction factors with a wide spectrum of antiviral activity against influenza A viruses (e.g. H1N1 viruses), West Nile virus, dengue virus, filoviruses, HIV-1, HCV, venezuelan equine encephalitis virus (VEEV), chikungunya virus (CHIKV), vesicular stomatitis virus (VSV) and even SARS-coronavirus [8], [13]–[18]. The main function of IFITM5 is associated with bone development in vertebrates [4], [19], [20]. IFITM6 seems to be involved in macrophage functions in tumor suppression [20]. To date, however, there is no information about the functions of IFITM7 and IFITM10.

Several antiviral restriction factors (e.g. APOBEC3G, Tetherin, and SAMHD1) have been demonstrated to evolve under positive selective pressure from viruses [21]–[29]. As important virus inhibitors, IFITM1, IFITM2 and IFITM3 may have also undergone a similar co-evolutionary process, such as other antiviral restriction factors do. Despite this connection, relationships between antiviral functions and adaptive evolution in IFITM family have seldom been reported and although previous reports had illustrated the phylogenetic history of IFITM family in some eukaryotic species [2], [30], [31], there has been no detailed information about IFITM genes in vertebrates. In this study, we performed detailed evolutionary analyses not only to test whether the primate IFITM genes evolved under positive selection throughout primate evolution, but also to unravel the evolutionary history of vertebrate IFITM family.

## Results

### IFITM Gene Repertoires in Vertebrates

To characterize the IFITM gene repertoires in vertebrates, we searched 27 vertebrate genome sequences with high genome coverage (≥6×) or representing the major evolutionary lineages in vertebrate phylogeny (such as opossum, lizard, platypus, *etc.*), using previously described IFITM sequences as queries. The taxa included ten non-mammalian vertebrates (five fishes: stickleback, tetraodon, medaka, fugu, zebrafish; one amphibian: frog; one non-avian reptile: lizard; three birds: chicken, turkey, zebra finch) and 17 mammals covering six primates (human, chimpanzee, gorilla, orangutan, macaque, marmoset), four glires (mouse, guinea pig, rat, and rabbit), five other mammals (tree shrew, cow, horse, dog and elephant), 1 metatherian (opossum) and 1 prototherian (platypus). We divided the newly identified genes into three types based on the following criteria: (i) functional genes, which contain full-length ORFs with intact CD225 domain and C-terminal TMDs; (ii) putative functional genes, which have CD225 domains but contain incomplete ORFs; and (iii) pseudogenes, which are sequences with pre-mature stop codons.

We identified a total of 286 IFITM-related sequences (Table 1). The number of IFITM genes varies considerably between mammals and non-mammalian vertebrates. A total of 27 functional genes were identified from 10 non-mammalian species, ranging from one gene in stickleback or tetraodon to six genes in frog. By contrast, 134 functional genes were identified from 17 mammalian species, ranging from one in platypus or rabbit to 26 in marmoset. Additionally, 14 putative functional genes including 13 in mammals and one in non-mammalian vertebrates were identified. Among the 10 non-mammalian and 17 mammalian species, 10 and 102 pseudogenes were identified, respectively. Interestingly, among 10 non-mammalian vertebrates, frog has the highest number (six functional genes and eight pseudogenes) of IFITM-related sequences. Among the 17 mammals, marmoset has the highest number (40) of IFITM-related sequences, of which 26 are functional genes. Human has the second highest number (29) of IFITM-related sequences, of which 18 are pseudogenes, the maximum in vertebrates.

**Table 1:** Numbers of genes and pseudogenes in vertebrate IFITM family.

	Species	Name	Functionalgenes	Putativelyfunctionalgenes	pseudogenes	Total	Version ofgenomicsequence
**Eutheria**	*Homo sapiens*	Human	11	0	18	29	*GRCh37*
	*Pan troglodytes*	Chimpanzee	7	0	4	11	*CHIMP2.1.4*
	*Gorilla gorilla*	Gorilla	9	2	5	16	*gorGor3.1*
	*Pongo abelii*	Orangutan	9	1	12	22	*PPYG2*
	*Macaca mulatta*	Macaque	11	0	11	22	*MMUL1*
	*Callithrix jacchus*	Marmoset	26	4	10	40	*C_jacchus3.2.1*
	*Tupaia belangeri*	Tree shrew	4	2	7	13	*TREESHREW*
	*Mus musculus*	Mouse	9	0	14	23	*NCBIM37*
	*Rattus norvegicus*	Rat	11	0	3	14	*RGSC3.4*
	*Cavia porcellus*	Guinea pig	5	0	0	5	*cavPor3*
	*Oryctolagus cuniculus*	Rabbit	1	1	1	3	*oryCun2*
	*Canis familiaris;*	Dog	7	1	8	16	*BROADD2*
	*Equus caballus*	Horse	5	1	0	6	*EquCab2*
	*Bos taurus*	Cow	8	0	0	8	*UMD3.1*
	*Loxodonta africana*	Elephant	5	0	4	9	*LoxAfr3*
**Metatheria**	*Monodelphis domestica*	Opossum	4	0	2	6	*BROADO5*
**Prototheria**	*Ornithorhynchus anatinus*	Platypus	1	1	3	5	*OANA5*
**Bird**	*Gallus gallus*	Chicken	3	0	1	4	*WASHUC2*
	*Meleagris gallopavo*	Turkey	3	0	0	3	*UMD2*
	*Taeniopygia guttata*	Zebra finch	2	0	0	2	*taeGut3.2.4*
**Reptile**	*Anolis carolinensis*	Anole lizard	3	0	1	4	*AnoCar 2.0*
**Amphibian**	*Xenopus tropicalis*	Frog	6	0	8	14	*JGI_4.2*
**Teleost**	*Takifugu rubripes*	Fugu	3	1	0	4	*Fugu4*
	*Oryzias latipes*	Medaka	3	0	0	3	*MEDAKA1*
	*Gasterosteus aculeatus*	Stickleback	1	0	0	1	*BROADS1*
	*Tetraodon nigroviridis*	Tetraodon	1	0	0	1	*TETRAODON8*
	*Danio rerio*	Zebrafish	2	0	0	2	*Zv9*
Total	27		160	14	112	286	

### Evolutionary Relationship of the Vertebrate IFITM Genes

**Figure 2:**
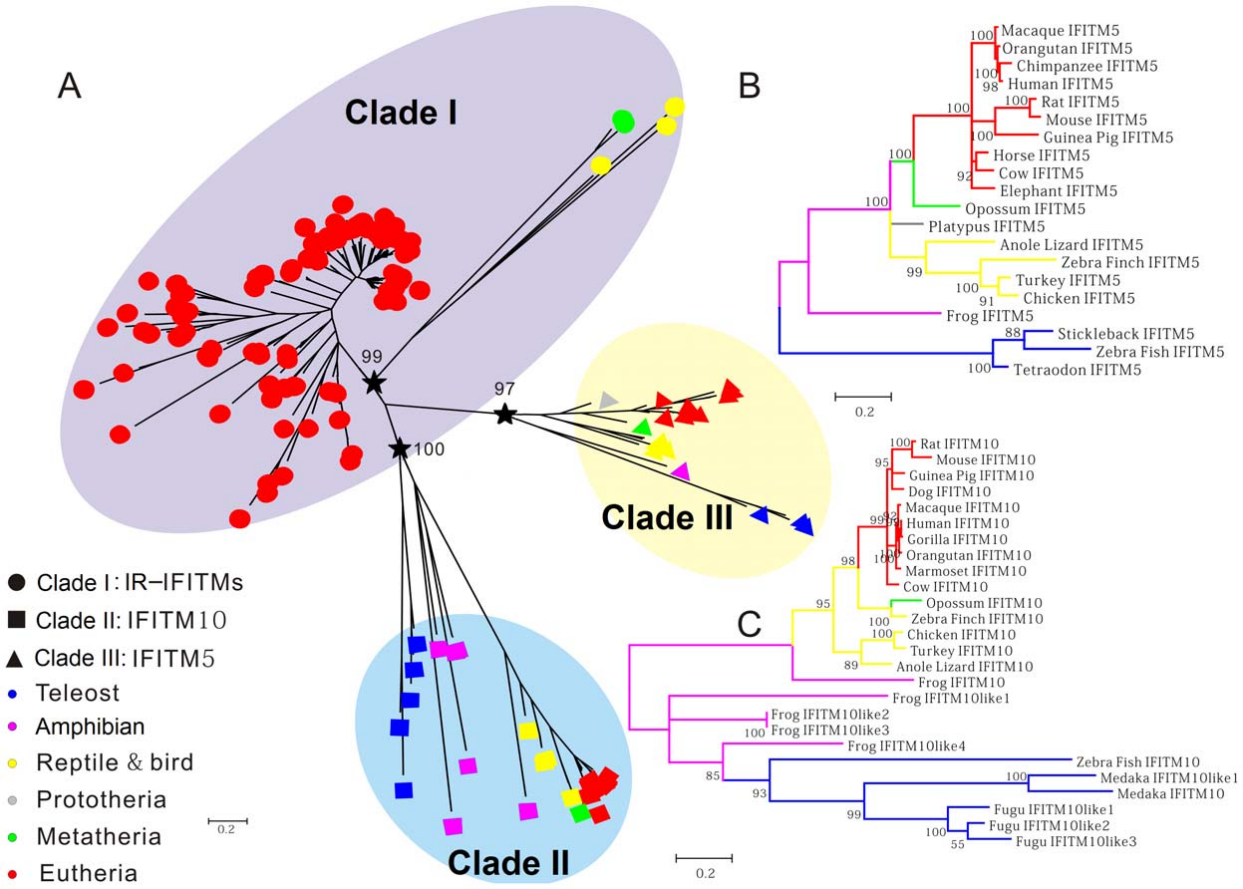
Bayesian tree of the vertebrate IFITM gene family. Bayesian tree was constructed by MrBayes v3.1.2 with 8 million generations. Branch confidence values are shown at the nodes. Scale bar corresponds to 0.2 substitutions per site. (**A**) Phylogenetic tree of all IFITM genes. (**B**) Sub-tree of IFITM5 genes. (**C**) Sub-tree of IFITM10 genes. Three stars represent 3 earlier progenitors of the IFITM family. Circle, square and triangle represent immunity-related IFITM, IFITM10 and IFITM5 genes, respectively. Different colors indicate different taxa.

To understand the phylogenetic relationship of IFITM gene family, 160 functional IFITM genes identified from 27 vertebrate species (Table S1) were subjected to phylogenetic analyses using Bayesian inference, maximum likelihood (ML) and maximum parsimony (MP) methods. The Bayesian, ML and MP trees show consistent topological structures (Figs. 2A and S1). All functional IFITM genes were well divided into three clades I, II and III with Bayesian posterior probabilities of ≥97. These analyses indicate that the IFITM family most likely originated from three progenitors.

The clades I, II and III contain 114, 26, and 20 IFITM genes, respectively. All IFITM10 and IFITM5 genes are clustered in clades II and III, respectively. All other functional IFITM genes, including IFITM1, IFITM2, IFITM3, IFITM6 and IFITM7, are grouped in clade I, forming the biggest sub-family in the IFITM family. Because the expression of the IFITM genes in clade I can be induced by IFN and their functions are associated with immunity [8], [13], [15], they are defined as immunity-related IFITM (IR-IFITM) sub-family.

In each clade, eutherian IFITM genes form a separate group from opossum and bird genes, consistent with the species phylogeny. Besides IFITM5 and IFITM10, IR-IFITM genes also have orthologs in both marsupials and eutherians, arguing against previous observation that only IFITM5 and IFITM10 orthologs could be identified in marsupials and eutherians [30]. In addition, clade II and III contain homologous IFITM sequences from teleosts and amphibians, but clade I does not (Fig. 2A), indicating that IR-IFITM originated later than IFITM5 and IFITM10.

### Phylogenetic Analyses of the Vertebrate Immunity-related IFITM Sub-family

**Figure 3:**
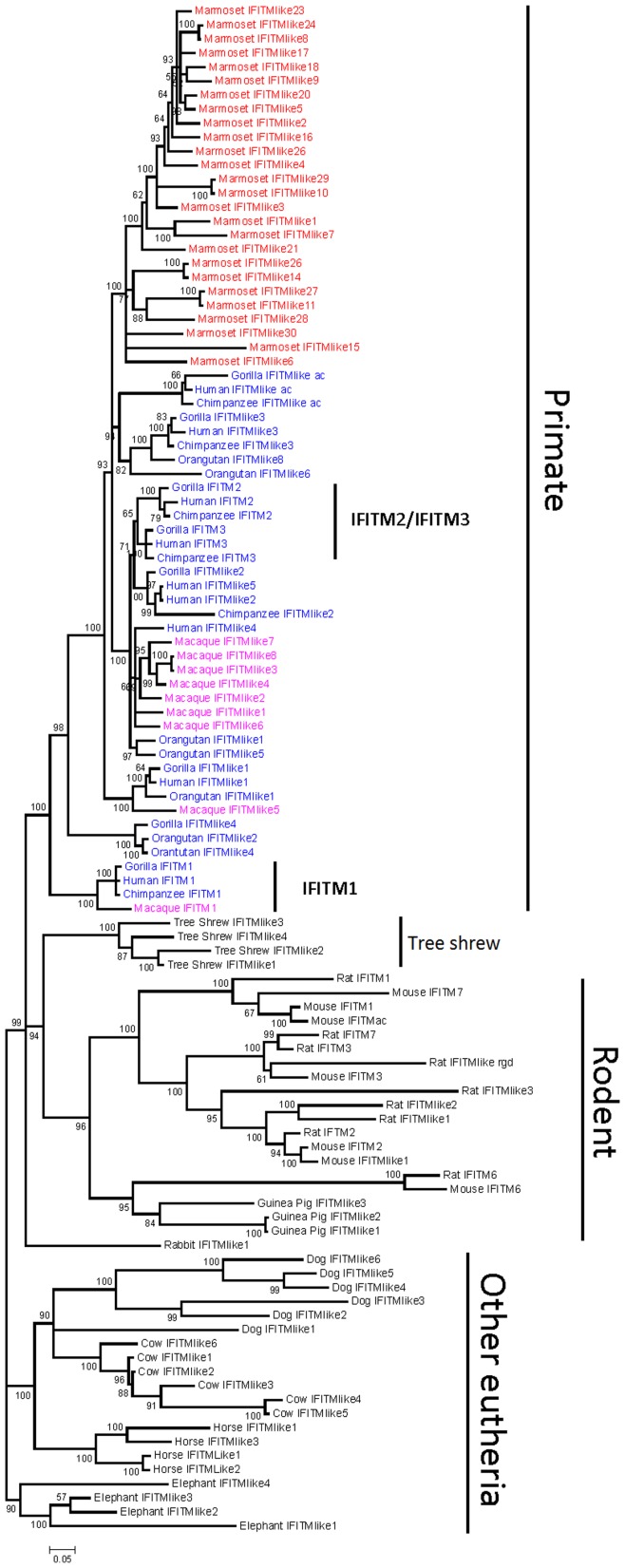
Bayesian tree of IR-IFITM genes in 14 eutheria species. Scale bar corresponds to 0.05 substitutions per site. Blue, purple and red represent hominids (human, chimpanzee, gorilla and orangutan), Old World monkey (macaque) and New World monkey (marmoset), respectively. Other species are shown in black.

IR-IFITM genes can be divided into two groups: one consisting of eutherian homologs and the other including homologs from metatheria and bird. We further constructed a sub-tree to show the phylogenetic relationship of 109 IR-IFITM genes from eutheria (Fig. 3). The sequences from elephant are located on the basal position. All IR-IFITM genes from the primate lineages form a sub-clade, and those from rodents form another sub-clade. Four genes from tree shrew form a species-specific cluster located between the sub-clades of the primates and the rodents. Three mammal species, dog, horse and cow, form several species-specific IR-IFITM gene clusters, which further compose a sub-clade in accordance with the phylogeny of these three species. These suggest that IR-IFITM genes evolved via gene duplication after species separation.

Interestingly, the majority of IR-IFITM genes from rodents do not form species-specific clusters as each of IFITM1, IFITM2 and IFITM3 clusters together in a lineage-specific manner. This clustering indicates that IR-IFITM genes have diverged into different IFITM isoforms prior to the split of rodents from other mammals. Additionally, in the lineage-specific clusters, more than one IR-IFITM gene was observed from certain species, indicating that gene duplication of IR-IFITM genes continued until after species separation of the rodents. Furthermore, rat IFITM7 clusters closely with rat IFITM3, indicating that they are a pair of duplicated genes. Similarly, mouseIFITM7, mouseIFITM1 and mouseIFITM-like3 (IFITMac) are another group of duplicated genes, suggesting that IFITM7 might have similar biological function to IFITM3 or IFITM1.

**Figure 4:**
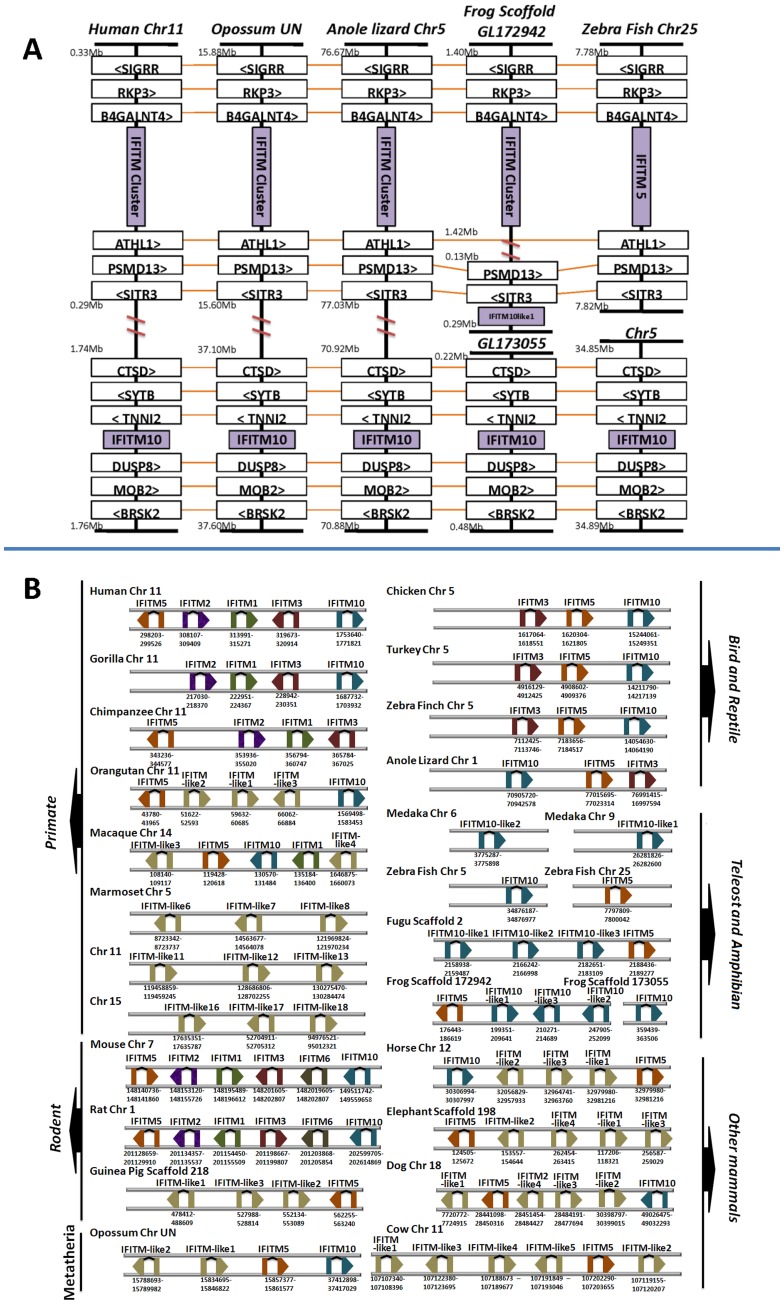
Syntenic context and chromosome location of the vertebrate IFITM genes. (**A**) Syntenic context and chromosome location of IFITM family. (**B**) Order and orientation of IFITM genes in the gene-cluster. Two-connected boxes represent two exons of each IFITM gene and the arrow indicates the orientation of transcription. Only chromosomes containing genes from IFITM gene-cluster and/or IFITM10 are shown in panel B.

Within the primate sub-clade, three separate clusters of IFITM1, IFITM2, and IFITM3 were observed. The IFITM1 cluster contains the sequences from all analyzed primates, excluding marmoset and orangutan, and is located at the basal position of the primate sub-clade, indicating that IFITM1 separated earlier than other IR-IFITM genes, including IFITM2 and IFITM3, during the primate evolution. The IFITM2 and IFITM3 clusters only contain sequences from three hominids (human, chimpanzee and gorilla), indicating that IFITM2 and IFITM3 originated prior to the separation of these three hominids. Likewise, similar to the rodent sub-clade, primate IR-IFITM genes also form different clusters, which contain orthologous IFITM genes from different species, indicating that most IR-IFITM members diverged prior to species separation. Furthermore, some IR-IFITM genes from same species cluster together and form species-specific sub-clusters, indicating that the IR-IFITM sub-family experienced a rapid expansion through gene duplications after the divergence of primates. Interestingly, a species-specific cluster was formed by the 25 IR-IFITM genes from marmoset, suggesting a rapid expansion of IR-IFITM genes by gene duplication. This speculation is supported by the four pairs of marmoset IFITM genes (IFITM-like8 and IFITM-like24, IFITM-like10 and IFITM-like29, IFITM-like14 and IFITM-like26, and IFITM-like11 and IFITM-like27) that exhibit very close genetic relationships, possibly indicating relatively recent gene duplication events. Additionally, the two genes in each pair are located in different chromosomes (Fig. 4B), indicating their origination by segmental duplication rather than tandem duplication.

### Phylogenetic Analyses of the Vertebrate IFITM10 and IFITM5 Sub-families

In total, 20 IFITM5 genes were identified in 20 vertebrates, covering species from teleosts to eutherians (Fig. 2B). No IFITM5 gene was identified in gorilla, marmoset, tree shrew, rabbit, dog, fugu, or medaka. Due to low sequencing coverage, we cannot rule out the possibility that the lack of IFITM5 in these genomes should be ascribed to relatively low quality of the genome sequences. The phylogenetic relationships of the available IFITM5 genes are consistent with the known species phylogeny. Only one IFITM5 gene was identified in each species, indicating that no gene duplication occurred in IFITM5 gene during the evolution of vertebrates.

In 19 vertebrates, 26 IFITM10 genes were identified, covering species from teleosts to eutherians (Fig. 2C). Each terrestrial vertebrate we surveyed possesses one IFITM10 gene, implying that no gene duplication occurred during the evolution of terrestrial vertebrates. In semi-aquatic frog, one IFITM10 and four IFITM10-like genes were identified. In aquatic vertebrates, three and two IFITM10 or IFITM10-like genes were identified in fugu and medaka, respectively. These indicate that species-specific gene duplications occurred in lower vertebrates. Within the IFITM10 clade, two groups were observed. One includes four IFITM10-like genes from frog and various numbers of IFITM10-like genes from teleosts. The other one comprises the frog IFITM10 gene and all IFITM10 genes from terrestrial vertebrates. Such division might distinguish aquatic- and terrestrial-type of IFITM10 and accordingly the amphibian frog possesses both types. These findings suggest that functional divergence of IFITM10 in the evolution of vertebrates may have occurred during the transition from an aquatic to a terrestrial environment.

### Conservation of Synteny in IFITM Genes during Vertebrate Evolution

In order to further understand the evolutionary scenario of IFITM family, we investigated the chromosomal distribution of well-defined IFITM genes including IFITM1, IFITM2, IFITM3, IFITM5, IFITM6, and IFITM10. Because IFITM7 has chromosome location independent from any other IFITM genes, and only mouse and rat have IFITM7 gene, we did not take it into account in this analysis. Orthologous relationships between IFITM family members were well confirmed with conserved syntenies (Fig. 4A). All these genes are located in one chromosome and form two loci in terrestrial vertebrates, except in cow that has two loci in two different chromosomes. With the exception of IFITM10, that is located at one locus, all other IFITM genes gather together and form a gene-cluster in the other locus. In teleosts (e.g. zebrafish), the two loci are dispersed in two different chromosomes. Although we identified two loci in two scaffolds of frog, whether they are dispersed in two different chromosomes is still unknown. We investigated genes flanking both sides of the two IFITM loci, and found that the two loci have almost completely same flanking genes from lower (e.g. zebrafish) to higher (e.g. human) vertebrate species. These findings suggest that chromosomal fusion might have occurred during the vertebrate evolution from aquatic to terrestrial species. Furthermore, all these IFITM genes have two exons, and the IFITM gene-clusters exhibit consistent gene order in four hominids, two rodents and three birds, supporting the syntenic relationship of IFITM genes.

The IFITM gene-clusters of different vertebrate species contain a variety of IFITM gene numbers (Fig. 4B). Due to incomplete genome information, the gene-cluster of some species including tree shrew, platypus and some teleosts are not determined. In zebrafish, the orthologous IFITM gene-cluster only contains IFITM5 in chromosome 25. In bird and lizard, two IFITM genes IFITM3 and IFITM5 are included in the gene-cluster. The hominid IFITM gene-clusters contain 3–4 IFITM genes, and the two rodent (mouse and rat) clusters contain five IFITM genes. In other mammals (cow, dog, horse, and elephant), gene-clusters have 4–6 IFITM or IFITM-like genes, including IFITM5 and various number of IFITM-like genes.

### Functional Divergence Among Different IFITM Gene Clades

**Figure 5:**
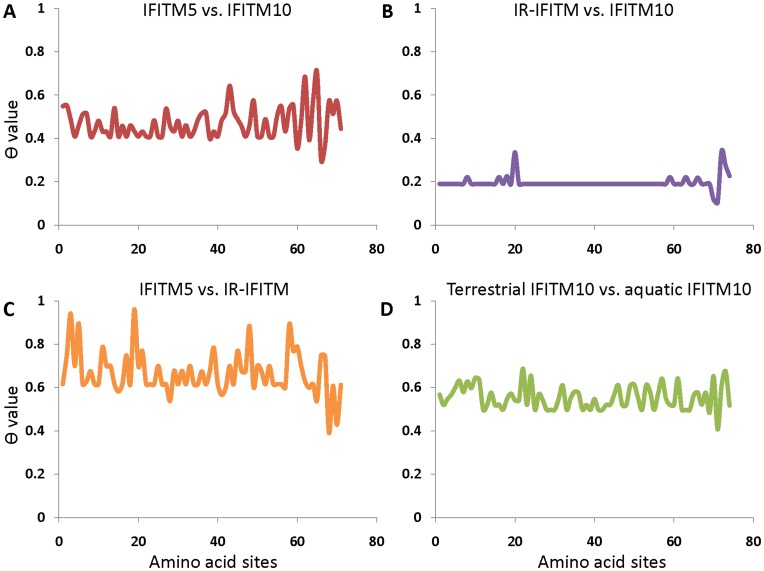
Functional divergence of the vertebrate IFITM families. Estimate of functional divergence was performed in the conservative region (CD225 domain). The X-axis stands for IFITM amino acids position and the Y-axis represents the values of θ, which indicates the level of functional divergence. (**A**) IFITM5 *vs.* IFITM10; (**B**) IFITM10 *vs.* IR-IFITM; (**C**) IFITM5 *vs.* IR-IFITM; (**D**) terrestrial IFITM10 *vs.* aquatic IFITM10.

The IFITM gene family was divided into three clades. Clade I was further divided into several sub-clades. To test whether there is functional divergence between different clades or between different sub-clades, we estimated type I divergence using DIVERGE v2.0 [32] and detected significant functional divergence between IR-IFITM and IFITM5, and between IFITM10 and IFITM5 (P<0.01). However, functional divergence signal was not detected between the IR-IFITM genes and IFITM10 (P = 0.0655) (Fig. 5 and Table 2). IFITM10 genes are divided into two sub-groups, terrestrial-type and aquatic-type and significant functional divergence was also detected between the two sub-groups (Fig. 5D). Among IR-IFITM genes, although IFITM2 and IFITM3 might originate from IFITM1 via gene duplication, there is no functional divergence observed between IFITM1 and IFITM2&3 genes (data not shown).

**Table 2:** Functional divergence of the vertebrate IFITM family.

	θ value		Standard error		P value
	MFE Method	MLE method	MFE method	MLE method	(MFE Method)
**IR-IFITM ** ***vs.*** ** IFITM5**	0.523	0.289	0.150493	0.279	<0.001
**IR-IFITM ** ***vs.*** ** IFITM10**	0.3926	0.368	0.274667	0.11328	0.0655
**IFITM5 ** ***vs.*** ** IFITM10**	0.726	0.765	0.282049	0.317	0.0028
**terrestrial IFITM10 ** ***vs.*** ** aquatic IFITM10**	0.537	0.564	0.136	0.227	<0.001

**θ,** coefficient of functional divergence; **MFE,** Model-Free Method; **MLE,** Maximum-Likelihood Estimation.

Conversely, crucial amino acid residues responsible for the functional divergence of IFITM genes among the three clades were predicted using a posterior-based site-specific profile (Fig. 5). Surprisingly, almost all sites of IFITM CD225 domains are crucial for the functional divergence between IR-IFITM and IFITM5. Some residues located in the CD225 domain and the C-terminal regions of IFITM protein are responsible for the functional divergence between IFITM5 and IFITM10.

### Positive Selection Acting on the Different IFITM Sub-families

To investigate whether positive selection drove the evolution of the vertebrate IFITM gene family, we calculated the non-synonymous substitution (dN) and synonymous substitution (dS) distances [33] between each pair of the sequences from the three clades. To exclude false signals caused by recombination, we first evaluated the effect of gene conversion using GENECONV [34]. Gene conversions were found in some species which are under species-specific duplication including dog, cow, horse, *etc.* (Table S2). Those sequences were removed from our datasets for subsequent analyses.

**Figure 6:**
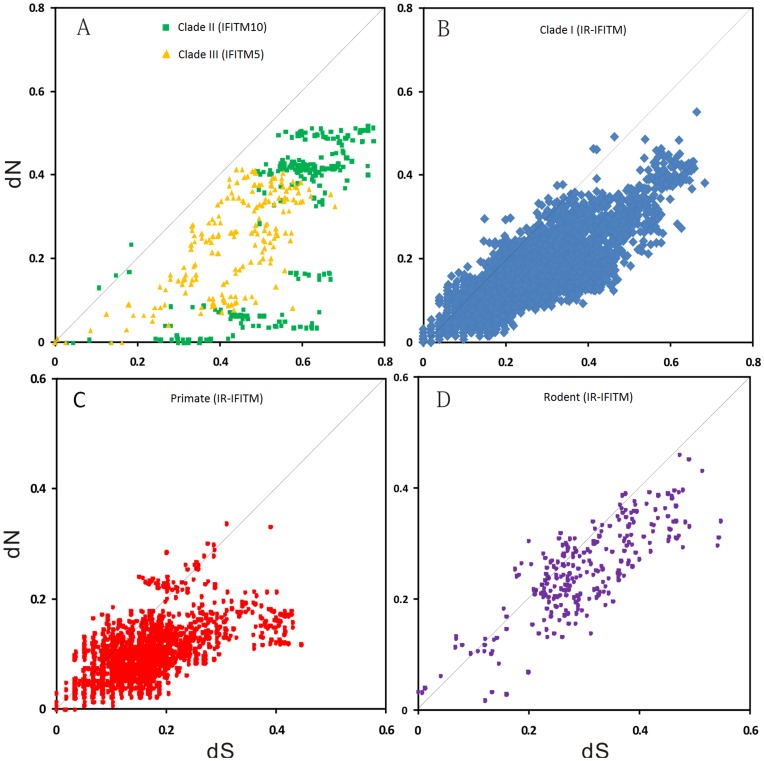
Pairwise comparisons of dN and dS for IFITM gene family. (**A**) clades II and III; (**B**) clade I; (**C**) the primate IR-IFITM genes; (**D**) the rodent IR-IFITM genes.

There is no significantly higher dN than dS in the pairwise comparisons of the sequences from clade III, suggesting that no positive selection acted on IFITM5 (Fig. 6A). Further site-specific analyses using PAML (Table 3) and HyPhy (data not shown) confirmed no positive selection acting on IFITM5 genes. There are three pairs of IFITM10 genes (in fugu: IFITM10-like3 *vs.* IFITM10-like2, and IFITM10-like1 *vs.* IFITM10-like2; in medaka: IFITM10-like1 *vs.* IFITM10-like2) with dN/dS>1 (Fig. 6A), indicating positive selection acting on aquatic IFITM10 gene. Because gene expansion of IFITM10 was observed in frog (Fig. 2C), we analyzed the five IFITM10 related genes (including one IFITM10 and four IFITM10-like genes) using the site-specific model. A strong signal of positive selection was detected. Eight sites were identified as under positive selection at the level of posterior probability>0.8 (Table 3). We further constructed the phylogeny of frog IFITM10-related genes and counted the numbers of non-synonymous (n) and synonymous (s) substitutions on each branch (Fig. S6). The ∑n/∑s ratio (1.75) is significantly smaller than the N/S ratio (2.96) (P = 0.0112, chi-square test), showing no positive selection. However, a very strong signal of positive selection (ω = 999, P = 0.0077, chi-square test) was observed on the branch leading to IFITM10-like1, -like2 and -like3 genes, indicating an episodic adaptive evolution.

**Table 3:** Site-specific tests for positive selection on different IFITM sub-families.

	Models	lnL	Estimates of parameters	2Δl	Positively selected sites
Clade I (IR-IFITM)	**The primate IR-IFITM**				
	M7 (beta)	−878.70	p = 0.122 q = 0.300	4.304 (P = 0.12)	NA
	M8 (beta & ω )	−876.55	p0 = 0.904 p = 0.567 q = 3.060, (p1 = 0.0964) ω = 2.54	4.304 (P = 0.12)	70P,133Y
	**The primate IFITM1**				
	M7 (beta)	−636.02	p = 44.67, q = 99.00	0.00 (P = 1.00)	NA
	M8 (beta & ω )	−636.02	p0 = 0.999, p = 44.67, q = 99.00, (p1 = 0.001), ω = 1	0.00 (P = 1.00)	None
	**The primate IFITM2&3**				
	M7 (beta)	−706.09	p = 0.019, q = 0.0.0584	0.02 (P = 0.99)	NA
	M8 (beta & ω )	−706.10	p0 = 0.780, p = 0.067, q = 1.935, (p1 = 0.220),ω = 1.00	0.02 (P = 0.99)	NS
	**Marmoset IR-IFITM**				
	M7 (beta)	−2062.37	p = 1.011, q = 1.069	8.70 (P = 0.014)	NA
	M8 (beta & ω )	−2058.02	p0 = 0.987, p = 1.118, q = 1.220, (p1 = 0.013),ω = 4.74	8.70 (P = 0.014)	16H**
	**The rodent IR-IFITM**				
	M7 (beta)	−2305.28	p = 0.866, q = 0.867	5.24 (P = 0.07)	NA
	M8 (beta & ω )	−2302.67	p0 = 0.935, p = 1.064, q = 1.241, (p1 = 0.065),ω = 1.88	5.24 (P = 0.07)	130V*
Clade II (IFITM10)	**The vertebrate IFITM10**				
	M7 (beta)	−2409.68	p = 0.649, q = 14.524	0.00 (P = 1.00)	NA
	M8 (beta & ω )	−2409.68	p0 = 1.000, p = 0.649, q = 14.524, (p1 = 0.000),ω = 1.00	0.00 (P = 1.00)	None
	**Frog IFITM10 related genes**				
	M7 (beta)	−1619.70	p = 0.68, q = 1.09	13.98 (P<0.01)	NA
	M8 (beta & ω )	−1612.71	p0 = 0.767, p = 1.575, q = 5.145, (p1 = 0.233),ω = 4.16	13.98 (P<0.01)	(2P, 11K, 22P, 35P, 36S, 79L, 112R, 116A)^a^
Clade III (IFITM5)	**The vertebrate IFITM5**				
	M7 (beta)	−2802.51	p = 0.733, q = 4.933	0.01 (P = 0.99)	NA
	M8 (beta & ω )	−2801.61	p0 = 0.980, p = 0.838, q = 6.388, p1 = (0.019), ω = 1.00	0.01 (P = 0.99)	None

lnL, the log-likelihood difference between the two models; 2Δl, twice the log-likelihood difference between the two models; NA, not allowed; NS, not shown (it means the sites under positive selection but not reaching the significance level of 0.9). The positively selected sites were identified with posterior probability ≥0.9 using Bayes empirical Bayes (BEB) approach. One asterisk indicates posterior probability ≥0.95, and two asterisks indicate posterior probability ≥0.99. Codon positions from top to bottom are according to the sequences of human IFITM3, human IFITM1, human IFITM3, marmoset IFITM-like1, mouse IFITM3, frog IFITM10, human IFITM10 and human IFITM5, respectively. Reference sequences can be found in Figure S5.

a the positively selected sites were identified with posterior probability ≥0.8.

Within clade I, 12 of 5996 pairwise comparisons exhibit significantly higher dN than dS (Fig. 6B and Table S3), indicating the presence of positive selection. Among 12 pairwise comparisons with significantly higher dN than dS, eight occur between the primate sequences (Fig. 5C), three between the rodent sequences (Fig. 5D), and one between cow sequences. Further site-specific analysis detected two positively selected sites (PSS) in the primate IR-IFITM genes and one in the rodent IR-IFITM genes (Table 3). No PSS was detected in the primate IFITM1 and IFITM2&3 subgroups. Because positive selection is generally associated with the occurrence of gene duplication [35], and a large number of gene duplications were observed in the marmoset IR-IFITM genes, we also performed site-specific analysis on marmoset IR-IFITM genes. As expected, one significant PSS was detected in marmoset IR-IFITM genes.

To confirm the results from PAML, similar positive selection analyses were performed using MEME on the DATAMONKEY server. The results reveal that there are five, four, seven and one sites underwent significant positive selection in primate, rodent, marmoset IR-IFITM groups and frog IFITM10 group, respectively (Table S4). PSSs identified by MEME method are consistent with those identified by PAML. Furthermore, positive selection can be also confirmed by branch-site REL method in both IR-IFITM and IFITM10 clades (Fig. S7). The major lineages that have undergone positive selection in the IR-IFITM clade are primate and rodent (Fig. S7A). Additionally, IFITM10 in lower vertebrate lineages also experienced strong selective pressure. Positively selected branches were detected in both teleost and frog lineages (Fig. S7B).

## Discussion

IFITM family contains seven members (IFITM1, IFITM2, IFITM3, IFITM5, IFITM6, IFITM7 and IFITM10), as well as some IFITM-like genes. All vertebrate IFITM genes are divided into three clades (Fig. 2A), implying origins from three progenitors. Clades I, II and III contain IR-IFITM, IFITM5 and IFITM10 genes, respectively. Substantial functional divergences occurred between IR-IFITM, IFITM5 and IFITM10 genes, indicating that IR-IFITM, IFITM5 and IFITM10 experienced individual evolutions. IR-IFITM, IFITM5 and IFITM10 genes are usually located in two loci (Fig. 4). One locus contains only the IFITM10 gene, and the other locus contains various numbers of IR-IFITM genes with a syntenic location with IFITM5, forming an IFITM gene-cluster. The two loci can be used as good markers to trace the evolutionary history of IFITM family. They are located in two different chromosomes in lower aquatic vertebrates, and evolved to lie in one chromosome by chromosomal fusion in higher mammals (Fig. 4A). The syntenic relationship of IR-IFITM genes and the presence of more IR-IFITM genes in mammals than other vertebrates suggest that IR-IFITM gene sub-family experienced a rapid expansion via tandem duplication during evolution from lower vertebrates to mammals.

Different IFITM members exhibit various functions. IFITM5 is specifically expressed in bone cells, but could not be induced by IFN stimulation [3], [19]. IFITM5 is involved in bone formation and considered as a bone-specific modulator of mineralization. The vertebrate IFITM5 genes form an independent clade in IFITM family (Fig. 2A). Neither gene duplication nor positive selection was identified in IFITM5 sub-family (Fig. 6B and Table 3), implying the functional conservation of IFITM5 in vertebrate evolution. Interestingly, no IFITM5 gene was found in the genomic data of two primate species, gorilla and marmoset. Gorilla has a close phylogenetic relationship with human and chimpanzee. In human and chimpanzee, IFITM5 is located upstream of the IFITM1-IFITM2-IFITM3 gene-cluster in chromosome 11. IFITM1, IFITM2 and IFITM3 in gorilla are also located in chromosome 11, and form a similar gene-cluster to those of human and chimpanzee (Fig. 4). Therefore, gorilla is also presumed to have an IFITM5 gene upstream of the IFITM1-IFITM2-IFITM3 gene-cluster. In fact, we found a region with incomplete sequencing at the corresponding locus of human or chimpanzee IFITM5 genes in chromosome 11 of gorilla, explaining why we could not identify IFITM5 gene locus in current gorilla genome dataset. In contrast, marmoset has special IFITM gene organization and experienced rapid gene expansion, thus forming unique species-specific gene cluster (Fig. 3). Considering that marmoset is one of the smallest monkeys in the world, as well as that IFITM5 plays a crucial role in bone formation [19], [36], we inferred that marmoset most likely lost its IFITM5 during the long period of evolution. Nevertheless, whether the loss of IFITM5 gene contributes to the tiny size of the marmoset needs to be determined by future studies.

So far, there is no study reporting the function of IFITM10. All various vertebrate IFITM10 genes can be divided into two sub-groups, one of which contains IFITM10 from terrestrial vertebrates with frog IFITM10 at the basal position, suggesting that terrestrial vertebrates and frog share a common IFITM10 ancestor. The other one contains IFITM10 from aquatic vertebrates, as well as four frog IFITM10-like genes. Significant signal of functional divergence was observed between the two sub-groups (Table 2), possibly suggesting an association with terrestrial and aquatic environments. In particular, both gene duplication and positive selection can be detected in IFITM10 or IFITM10-like genes from the aquatic vertebrates (Fig. 6D and Table 3), indicating that IFITM10 is associated with the adaptation to aquatic environments. There are one IFITM10 gene and four IFITM10-like genes in frog (Fig. 2C). An episodic adaptive evolution was found on the branch leading to three frog IFITM10-like genes (Fig. S6), supporting the association of IFITM10 with terrestrial and/or aquatic environments. However, what the function of IFITM10 is and how it helps the aquatic vertebrates to adapt to aquatic environments still need to be determined.

Distinct from IFITM5 and IFITM10, the IR-IFITM sub-family contains IFITM1, IFITM2, IFITM3, IFITM6, IFITM7, as well as a large number of IFITM-like genes [37], [38]. This sub-family forms a large clade in the phylogenetic tree (Fig. 3). The IR-IFITM genes from same mammalian species, such as dog, horse, cow, elephant, guinea pig and tree shrew, cluster together and form species-specific IR-IFITM gene sub-clusters (Fig. 3), indicating that gene duplication occurred after the separation of these mammalian species in a species-specific pattern. The same IFITM member from mouse and rat cluster together, indicating that the gene duplication there occurred prior to the separation of the two species in a lineage-specific pattern. The rodent IFITM1, IFITM2 and IFITM3 sub-groups cluster together (Fig. 3), suggesting a close phylogenetic relationship and similar functions. IFITM6 and IFITM7 are specific for the rodents. The rat and mouse IFITM6 genes cluster together and further group with the guinea pig IR-IFITM gene sub-cluster. Mouse IFITM7 clusters closely with mouse IFITM1 gene and rat IFITM7 clusters closely with rat IFITM3 gene (Fig. 3), which might indicate IFITM7 has similar functions to IFITM1 or IFITM3.

Lineage-specific gene duplications were also observed in the primate IR-IFITM genes. The IFITM1 genes from some primate species form an individual sub-group located at the basal position of the primate IR-IFITM sub-clade (Fig. 3). IFITM1, IFITM2 and IFITM3 have miscellaneous functions including cell adhesion, antiproliferation, tumor suppression and embryonic development. Apart from these biological functions, human IFITM1, IFITM2 and IFITM3 have a broad-spectrum of antiviral activity, possibly brought on by inhibiting the viral entry processes [14], [18], [39]. In particular, human IFITM3 and IFITM2 appear to have higher antiviral activity than IFITM1 [14] and IFITM3 has been reported to inhibit virus replication in other mammals [40]. These findings suggest that after IFITM1 occurrence, the generation of IFITM2 and IFITM3 might be associated with host defense against various virus infections.

Primate and rodent IFITM1, IFITM2 and IFITM3 have similar functions [2], but do not form a monophyletic cluster (Fig. 3), indicating that they do not share the most recent common ancestor (MRCA) and moreover suggesting convergent evolution of IFITM1, IFITM2 and IFITM3 in primates and rodents. Convergently evolved amino acids between primates and rodents were found in the C-terminus of IFITM2 and IFITM3 (data not shown), a crucial region for antiviral activity, supporting the association between viral infections and the evolution of IR-IFITM genes.

The largest scale of gene expansion of IR-IFITM genes was observed among the primates in a complex pattern that includes both lineage- and species-specific gene duplication events. The species-specific pattern mainly seems to have occurred in macaque and marmoset, giving rise to seven IFITM2&3-like genes in macaque and 29 IR-IFITM genes in marmoset (Fig. 3). Large scale of duplication and pseudogenization events suggest that the IR-IFITM clade evolved under birth-and-death model [41], [42]. Interestingly, however, marmoset does not possess any one of IFITM1, IFITM2 or IFITM3, and macaque possesses IFITM1 but not IFITM2 and IFITM3. Why marmoset and macaque evolved so many IR-IFITM genes remains unclear and should be explored in more detail in future studies.

The Red Queen hypothesis presumes that the antagonistic co-evolutionary dynamics of host-virus systems can generate selection for accelerated evolution of host antiviral restriction factors, just like the observations on the primate antiviral restriction factors APOBEC3G, Tetherin, and SAMHD1 [21]–[29]. We detected positive selection acting on marmoset IR-IFITM genes (Table 3). Macaque and marmoset are susceptible to infection by many contemporary viruses and are often used as suitable non-human primate models for viral infectious disease studies [9], [43]–[58]. Cell lines (e.g. kidney cells) from marmoset can be infected by most primate viruses, such as flaviviridae family of viruses (GBV-B), lassa virus, peste des petitis ruminants (PPR) virus and so on [59], [60], [61]. These imply that marmoset and macaque might be able to be infected by the ancestors of contemporary primate viruses and/or some unknown viruses during early evolution of primates. Additionally, relative to pig IFITM3 that has been demonstrated to have an antiviral activity [40], IFITM1, IFITM2 and IFITM3 genes from macaque and marmoset have higher similarity to human IFITM1, IFITM2 and IFITM3 in sequence and domain organization. These imply that although lacking experimental support, the IR-IFITM genes from macaque and marmoset might have similar antiviral activity to human IFITM1, IFITM2 and IFITM3. Therefore, the rapid expansion of IR-IFITM genes might be ascribed to the infection of marmosets by viruses.

In this study, we demonstrated the evolutionary dynamics of IFITM genes that diversifies in different sub-clades, probably in accordance with their distinct functions. Future studies on immunology, developmental biology and comparative biology to determine IFITM functions would better clarify the relationship between divergence and functions and likewise extend our knowledge on IFITM function and evolutionary mechanisms.

## Materials and Methods

### Sequences Data Collection

Functional IFITM gene sequences were gained based on orthologous and paralogous relationships by querying the Ensembl genome assemblies (http://www.ensembl.org/index.html) using known authentic IFITM genes [17]. Collected IFITM genes were used as queries to search against known IFITM gene datasets using tBLASTn or BLASTn searches to make sure the best hit is an functional IFITM genes with E value<10^−10^. The presence of CD225 domain in each obtained IFITM protein sequence was confirmed using P-fam database (http://pfam.sanger.ac.uk).

### Identification of Putative IFITM Functional Genes and Pseudogenes

Annotated IFITM genes were retrieved from Ensembl databases (http://www.ensembl.org/). To identify additional putative IFITM functional genes and pseudogenes, tBLASTn searches were performed in Ensembl using human IFITM1, IFITM2, IFITM3 and IFITM5 as queries against the available genome sequences of species listed in Table 1 with E value<10^−5^ [62]. After deleting the redundancies and merging overlapping sequences, the remained sequences with>150 nt were analyzed by GENSCAN to identify the putative coding sequence. Each candidate IFITM gene was used as a query to BLAST against GenBank non-redundant protein database to make sure the best hit is an IFITM gene. The exons and intron of the remained sequences were detected with GeneWise. The presences of CD225 domain and C-terminal TMD in candidate sequences were identified using the online tools SMART (http://smart.embl-heidelberg.de) and SOSUI (http://bp.nuap.nagoya-u.ac.jp/sosui/), respectively. If the sequence did not have complete CD225 domain and the C-terminal TMD, or its open reading frame (ORF) was disrupted, this sequence was regarded as a pseudogene. To avoid the possible error in pseudogene identification, the PSEDOPIPE approach (http://pseudofam.pseudogene.org/pages/main/about.jsf) [63] was further used to confirm identified IFITM pseudogenes. On the other hand, because authentic IFITM proteins are in a range of 102 to 157 amino acids [2], the candidates that are outside above range and have complete open reading frame, complete CD225 domain and the C-terminal TMD were identified as functional genes and referred to as IFITM-like genes.

Conserved motifs in the complete amino acid sequences of the mammalian IFITM proteins were analyzed by MEME/MAST software (http://meme.sdsc.edu/meme/website/intro.html). The secondary structure of IFITM proteins was predicted using SMART (http://smart.embl-heidelberg.de/smart). The logo pictures were generated by Weblogo (http://weblogo.berkeley.edu).

### Molecular Phylogenetic Analyses

Multiple sequence alignments were performed using Muscle in MEGA 5.0 [64] and were refined manually in Bioedit (http://www.mbio.ncsu.edu/BioEdit/BioEdit.html). Unambiguously aligned positions (Figs. S2, S3 and S4) were used for subsequent phylogenetic analyses. Maximum likelihood (ML) tree of IFITM gene family was reconstructed by PHYML2.4 implemented in Jmodeltest 0.1 package with the best-fitting model of F81+I+G that is selected using JmodelTest in the same package [65], [66]. The bootstrap analysis was performed with 1000 replications. Bayesian inference (Bayes) tree was reconstructed using MrBayes v3.1.2 [67]. Four independent Markov Chain Monte Carlo (MCMC) chains were used with the default temperature of 0.01. Four repetitions were run for 8,000,000 generations with tree and parameter sampling occurring every 1,000 generations. The first 25% of trees were discarded as burn-in, leaving 750 trees per run. Posterior probabilities for internal node were calculated from the posterior density of trees. Maximum parsimony (MP) tree was reconstructed by PAUP 4.0 with a bootstrap value of 1,000 repetitions [68]. To investigate whether gene conversion occurred in mammalian IR-IFITM genes, an analysis was performed using the GENECONV program [34].

### Positive Selection Analyses

To detect whether positive selection acted on IFITM family, the CODEML program implemented in PAML 4.2 package was used. The site-specific model was performed by comparing the models M2a (positive selection) and M8 (beta & ω) *vs.* the null models M1a (nearly neutral) and M7 (beta), respectively. Likelihood ratio tests (LRT) of different models were used to find the best fit model for the data [65], [66], [69]. We also used the MEME method and the branch-site REL model implemented in DATAMONKEY (http://www.datamonkey.org/) to confirm the results by PAML analyses [70], [71]. MEME is the latest method to identify PSS and can find signatures of episodic selection even when the majority of lineages are subject to purifying selection.

### Functional Divergence Analysis

In the phylogenetic tree, IR-IFITM, IFITM5 and IFITM10 genes from different species form three independent clusters. We investigated type I functional divergence among IR-IFITM, IFITM5 and IFITM10 using Diverge v2.0 with the Maximum-Likelihood Estimation (MLE) and Model-Free Method (MFE) [32]. Type I sites represent amino acids conservation in one cluster, but high variability in another, suggesting that these residues have been subjected to different functional constraints. The coefficient of functional divergence, θ, ranging from 0 to 1, was used to test the statistical significance of functional divergence that has occurred between different clusters. A null hypothesis of θ = 0 indicates that the evolutionary rate is virtually the same between two clusters at each site. When θ>0.5, the null hypothesis is considered to be significantly rejected. The important amino acid residues most likely to be responsible for functional divergence were then predicted by calculating the site-specific profile based on posterior analysis for all pairs of clusters with functional divergence.

## Supporting Information

Figure S1 **ML (A) and MP (B) trees of the vertebrate IFITM family.** ML and MP trees were constructed using PHYML v2.4 and PAUP 4.0, respectively. Bootstrap tests were performed with 1,000 replications. For other details, see Fig. 2.
(PDF)

Figure S2 **Sequence alignment of the vertebrate IFITM5 genes.** Alignment was used to reconstruct the Bayesian tree in Figure 2B.
(PDF)

Figure S3 **Sequence alignment of the vertebrate IFITM10 genes.** Alignment was used to reconstruct the Bayesian tree in Figure 2C.
(PDF)

Figure S4 **Sequence alignment of the mammalian IR-IFITM genes.** Alignment was used to reconstruct the phylogenetic trees shown in Figure 3. The abbreviations were adopted from the species names in Table 1.
(PDF)

Figure S5 **Proteins sequence alignments of IFITM5, IFITM10 and IR-IFITM genes.** Consensus (identical) amino acids are shown in the bottom line (Similarity Groups: 1, DN; 2, EQ; 3, ST; 4, KR; 5, FYW; 6, LIVM). Black or gray shading indicates conservation and similarity, respectively.
(PDF)

Figure S6 **Numbers of non-synonymous (n) and synonymous (s) substitutions in frog IFITM10 and IFITM10-like genes.** Actual numbers of n/s changes and ω values (dN/dS, in parentheses) are shown above each branch. N and S are the potential numbers of non-synonymous and synonymous sites, respectively. Red line represents the branch under positive selection.
(TIFF)

Figure S7 **Positive selection analyses of IFITM genes with branch-site REL model in DATAMONKEY.** Red lines indicate branches under positive selection. (**A**) Positive selection in primate and rodent IR-IFITM dataset. (**B**) Positive selection in vertebrate IFITM10 dataset.
(PDF)

Table S1 **Functional IFITM genes information used in this study.**
(XLS)

Table S2 **Gene conversion analyses of IR-IFITM genes.**
(DOC)

Table S3 **Twelve pairwise comparisons showing significantly higher dN than dS for IR-IFITM genes.**
(DOC)

Table S4 **Positively selected sites in IR-IFITM genes detected using MEME method implemented in DATAMONKEY.**
(DOC)
