# Supplementary material for: Evolutionary Dynamics of the Interferon-Induced Transmembrane Gene Family in Vertebrates
Source: PLoS One. 2012 Nov 15;7(11):e49265. doi: 10.1371/journal.pone.0049265 (PMC3499546; doi:10.1371/journal.pone.0049265)
Supplement: Figure S1 — ML (A) and MP (B) trees of the vertebrate IFITM family. ML and MP trees were constructed using PHYML v2.4 and PAUP 4.0, respectively. Bootstrap tests were performed with 1,000 replications. For other details, see Fig. 2. (PDF) [file pone.0049265.s001.pdf]

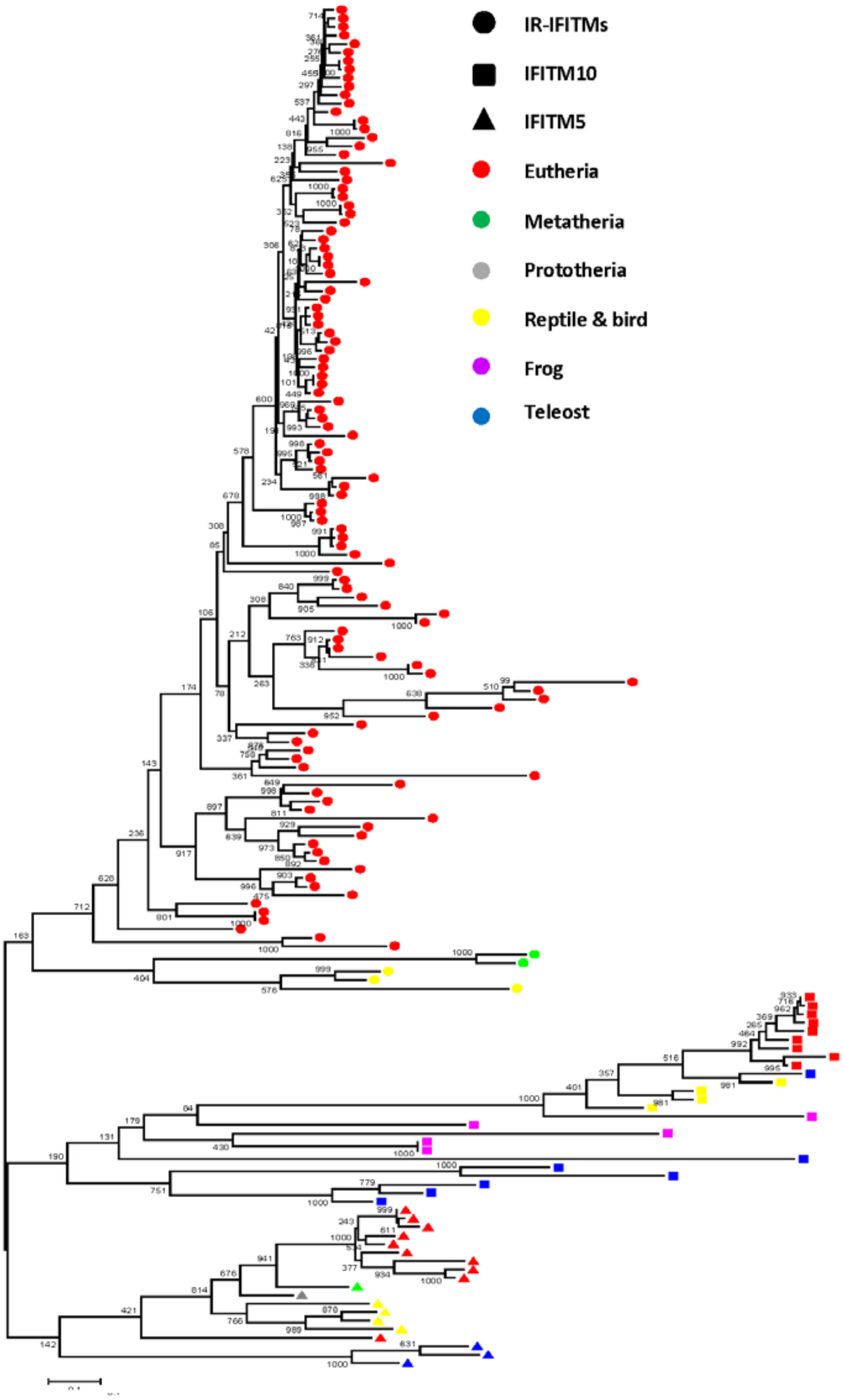
IR-IFITMs
IFITM10
IFITM5
Eutheria
Metatheria
Prototheria
Reptile & bird
Frog
Teleost

A

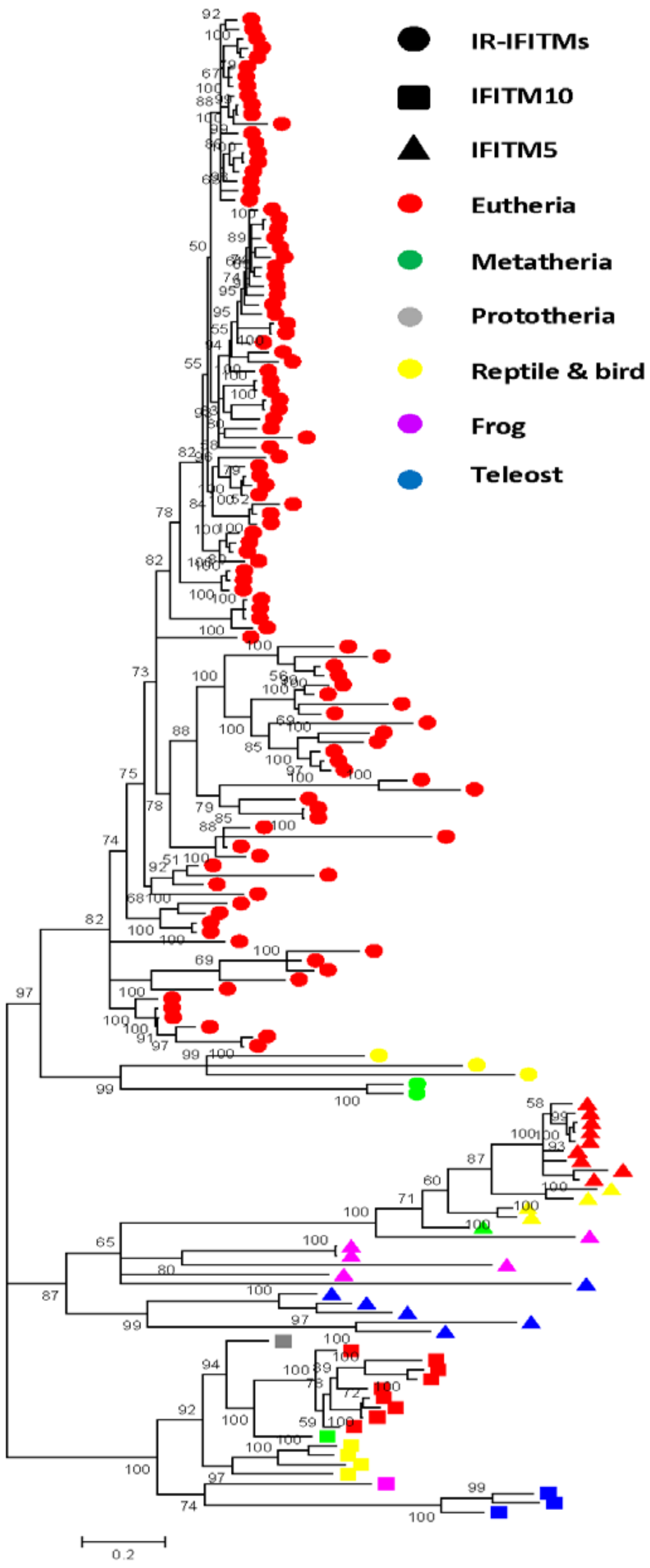

B

Figure S1. ML (A) and MP (B) trees of the vertebrate IFITM family. The ML and MP trees were constructed using PHYML v2.4 and PAUP 4.0, respectively. Bootstrap tests were performed with 1,000 replications. For other details, please see Fig. 2.
