# Supplementary material for: Evolutionary Dynamics of the Interferon-Induced Transmembrane Gene Family in Vertebrates
Source: PLoS One. 2012 Nov 15;7(11):e49265. doi: 10.1371/journal.pone.0049265 (PMC3499546; doi:10.1371/journal.pone.0049265)
Supplement: Figure S5 — Proteins sequence alignments of IFITM5, IFITM10 and IR-IFITM genes. Consensus (identical) amino acids are shown in the bottom line (Similarity Groups: 1, DN; 2, EQ; 3, ST; 4, KR; 5, FYW; 6, LIVM). Black or gray shading indicates conservation and similarity, respectively. (PDF) [file pone.0049265.s005.pdf]

IFITM5

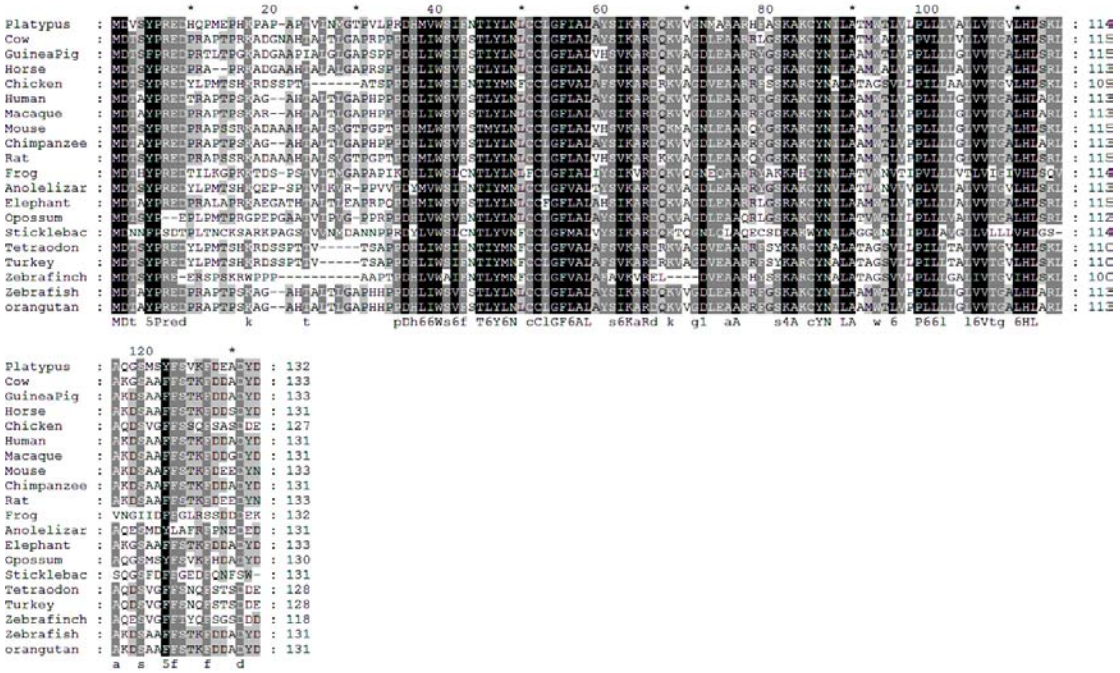

IFITM10

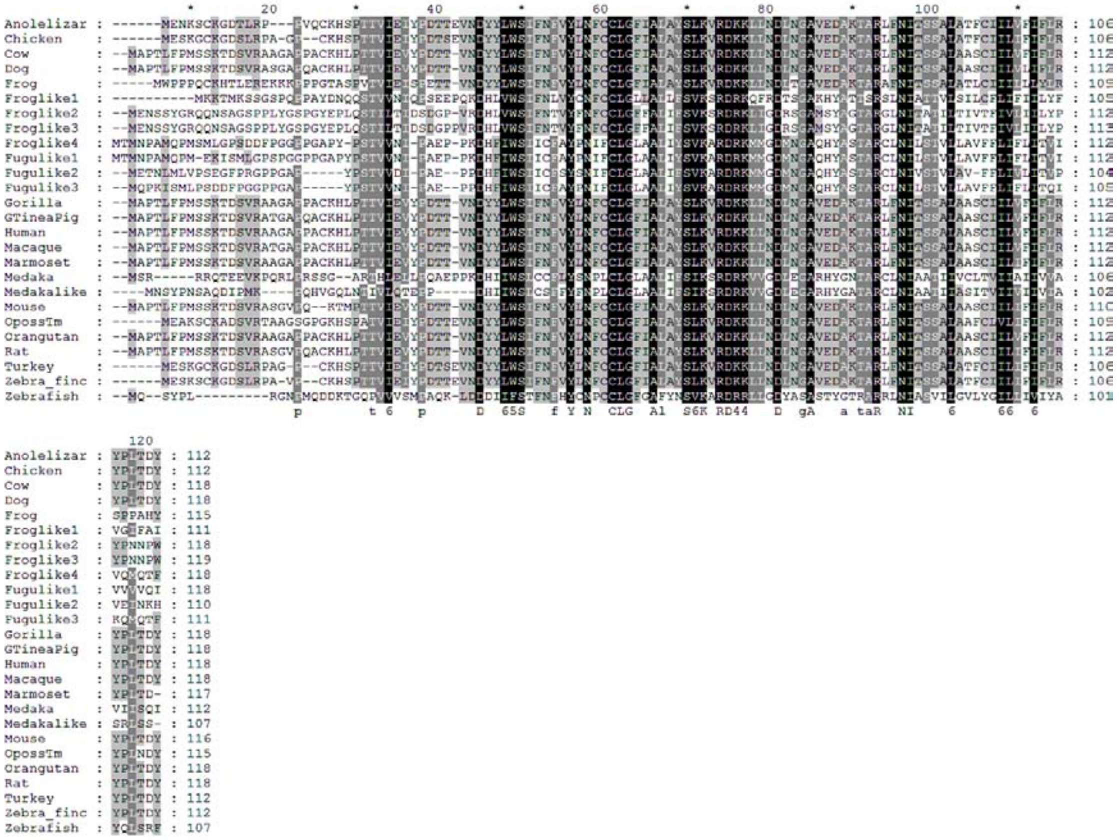

IR-IFITM

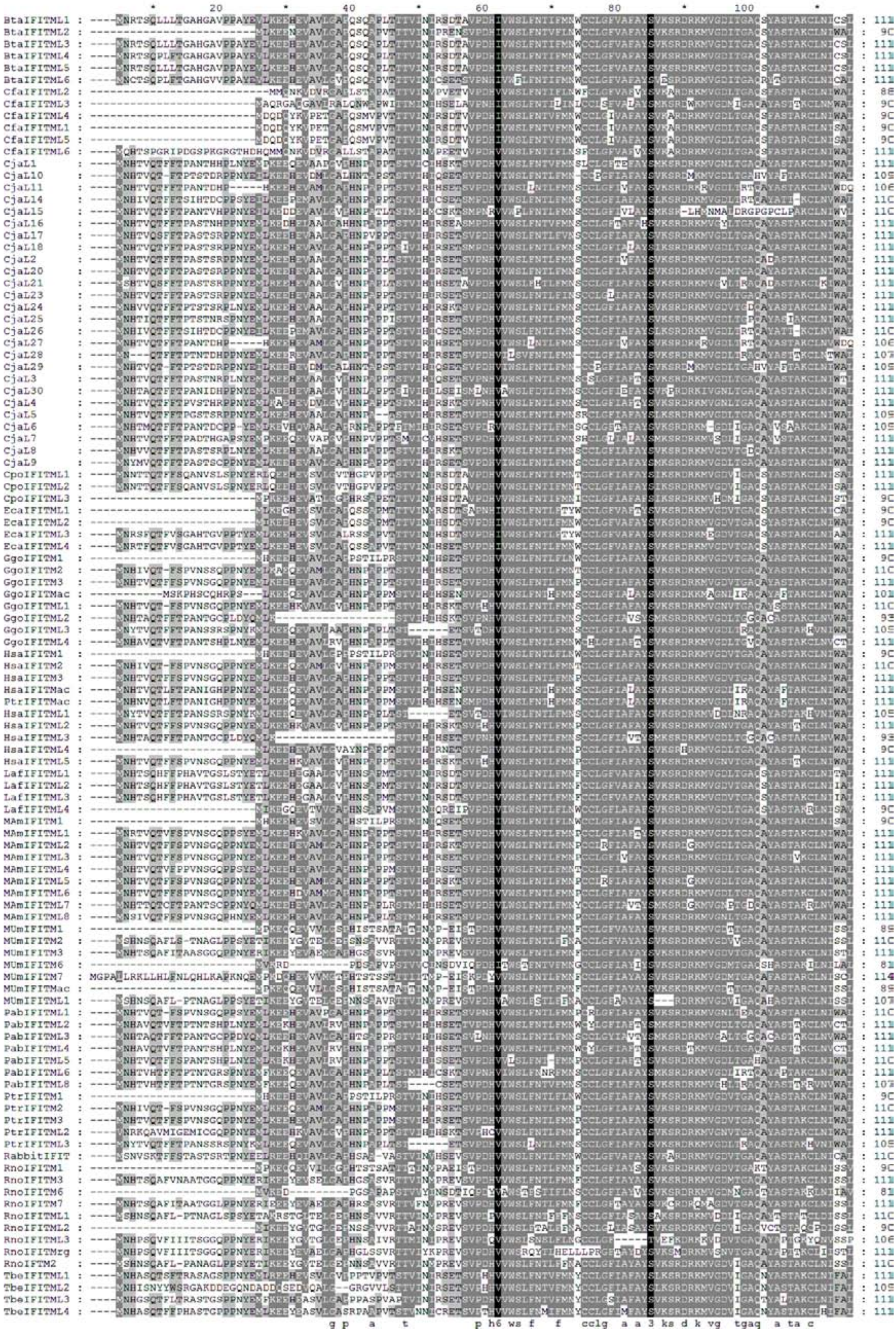

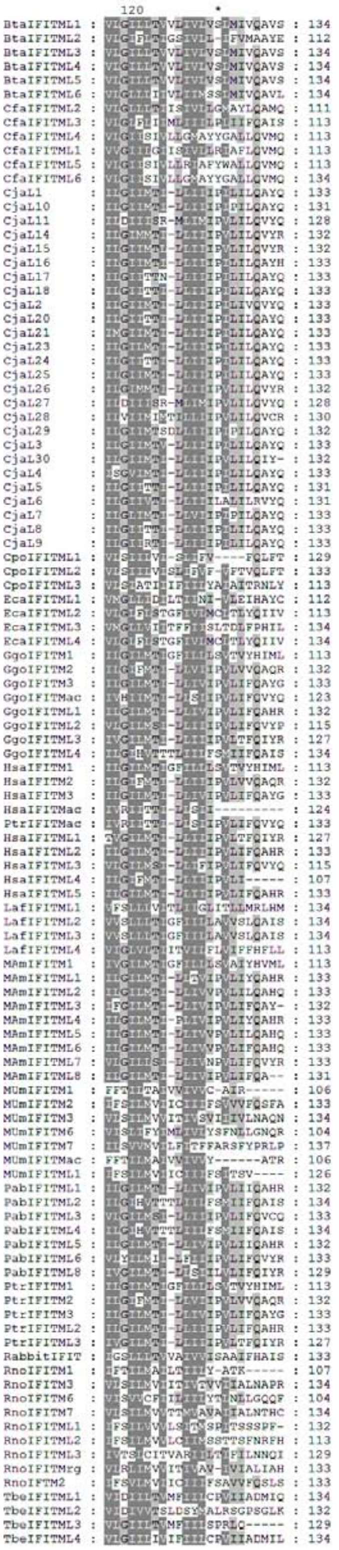

**Figure S5. Proteins sequence alignments of IFITM5, IFITM10 and IR-IFITM genes.** Consensus (identical) amino acids are shown in the bottom line (Similarity Groups: 1, DN; 2, EQ; 3, ST; 4, KR; 5, FYW; 6, LIVM). Black or gray shading indicates conservation and similarity, respectively.
