# Supplementary figures and images for: Evolutionary Dynamics of the Interferon-Induced Transmembrane Gene Family in Vertebrates
Source: PLoS One. 2012 Nov 15;7(11):e49265. doi: 10.1371/journal.pone.0049265 (PMC3499546; doi:10.1371/journal.pone.0049265)

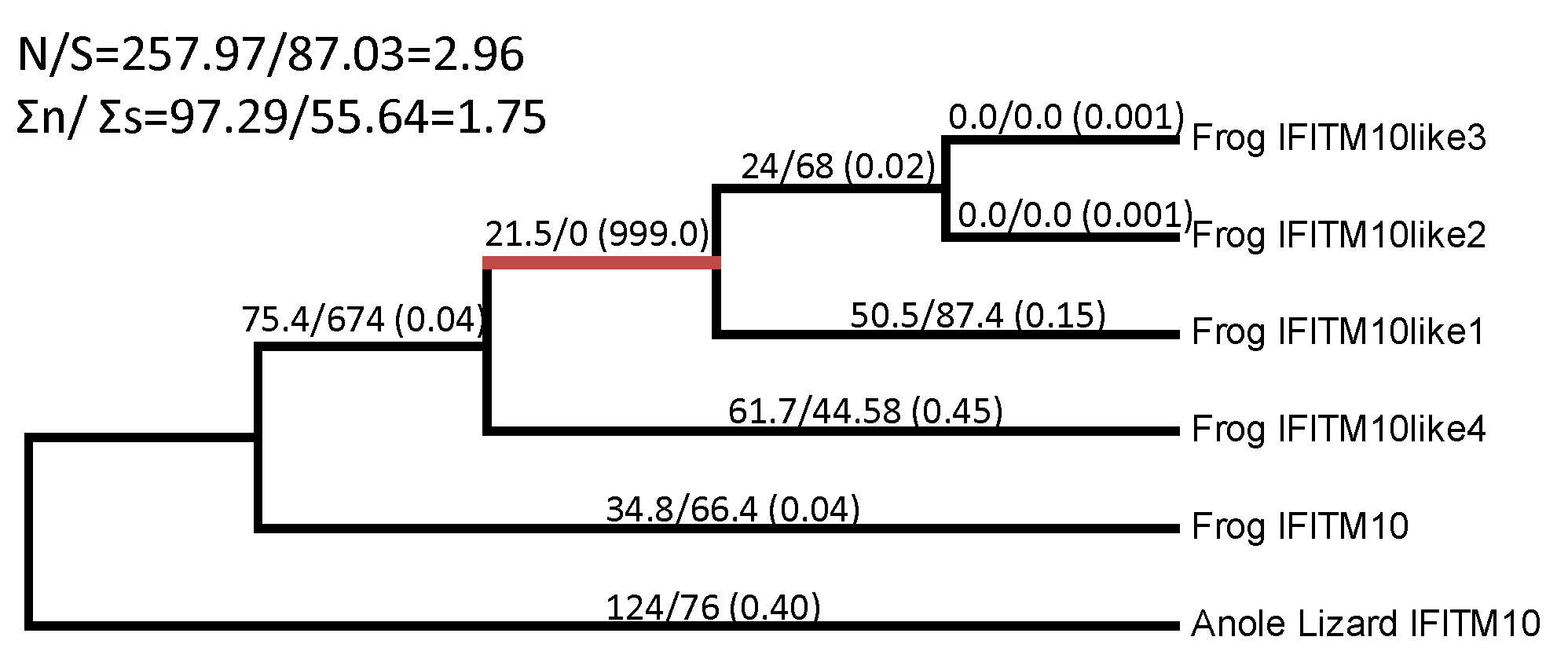

Supplement: Figure S6 — Numbers of non-synonymous (n) and synonymous (s) substitutions in frog IFITM10 and IFITM10-like genes. Actual numbers of n/s changes and ω values (dN/dS, in parentheses) are shown above each branch. N and S are the potential numbers of non-synonymous and synonymous sites, respectively. Red line represents the branch under positive selection. (TIFF) [file pone.0049265.s006.tiff]
