# Supplementary material for: Evolutionary Dynamics of the Interferon-Induced Transmembrane Gene Family in Vertebrates
Source: PLoS One. 2012 Nov 15;7(11):e49265. doi: 10.1371/journal.pone.0049265 (PMC3499546; doi:10.1371/journal.pone.0049265)
Supplement: Figure S7 — Positive selection analyses of IFITM genes with branch-site REL model in DATAMONKEY. Red lines indicate branches under positive selection. (A) Positive selection in primate and rodent IR-IFITM dataset. (B) Positive selection in vertebrate IFITM10 dataset. (PDF) [file pone.0049265.s007.pdf]

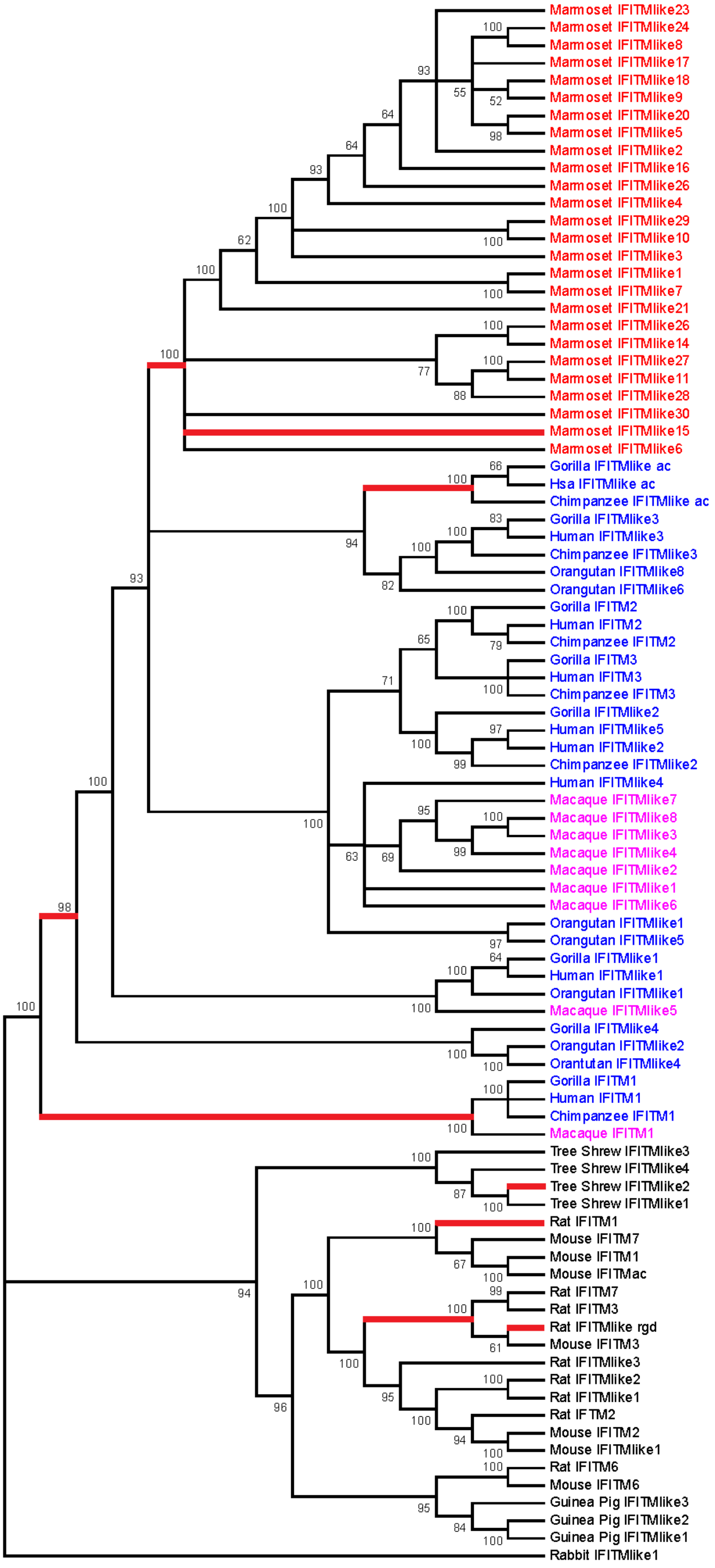
Marmoset IFITMlike23
Marmoset IFITMlike24
Marmoset IFITMlike8
Marmoset IFITMlike17
Marmoset IFITMlike18
Marmoset IFITMlike9
Marmoset IFITMlike20
Marmoset IFITMlike5
Marmoset IFITMlike2
Marmoset IFITMlike16
Marmoset IFITMlike26
Marmoset IFITMlike4
Marmoset IFITMlike29
Marmoset IFITMlike10
Marmoset IFITMlike3
Marmoset IFITMlike1
Marmoset IFITMlike7
Marmoset IFITMlike21
Marmoset IFITMlike26
Marmoset IFITMlike14
Marmoset IFITMlike27
Marmoset IFITMlike11
Marmoset IFITMlike28
Marmoset IFITMlike30
Marmoset IFITMlike15
Marmoset IFITMlike6
Gorilla IFITMlike ac
Hsa IFITMlike ac
Chimpanzee IFITMlike ac
Gorilla IFITMlike3
Human IFITMlike3
Chimpanzee IFITMlike3
Orangutan IFITMlike8
Orangutan IFITMlike6
Gorilla IFITM2
Human IFITM2
Chimpanzee IFITM2
Gorilla IFITM3
Human IFITM3
Chimpanzee IFITM3
Gorilla IFITMlike2
Human IFITMlike5
Human IFITMlike2
Chimpanzee IFITMlike2
Human IFITMlike4
Macaque IFITMlike7
Macaque IFITMlike8
Macaque IFITMlike3
Macaque IFITMlike4
Macaque IFITMlike2
Macaque IFITMlike1
Macaque IFITMlike6
Orangutan IFITMlike1
Orangutan IFITMlike5
Gorilla IFITMlike1
Human IFITMlike1
Orangutan IFITMlike1
Macaque IFITMlike5
Gorilla IFITMlike4
Orangutan IFITMlike2
Orantutan IFITMlike4
Gorilla IFITM1
Human IFITM1
Chimpanzee IFITM1
Macaque IFITM1
Tree Shrew IFITMlike3
Tree Shrew IFITMlike4
Tree Shrew IFITMlike2
Tree Shrew IFITMlike1
Rat IFITM1
Mouse IFITM7
Mouse IFITM1
Mouse IFITMac
Rat IFITM7
Rat IFITM3
Rat IFITMlike rgd
Mouse IFITM3
Rat IFITMlike3
Rat IFITMlike2
Rat IFITMlike1
Rat IFTM2
Mouse IFITM2
Mouse IFITMlike1
Rat IFITM6
Mouse IFITM6
Guinea Pig IFITMlike3
Guinea Pig IFITMlike2
Guinea Pig IFITMlike1
Rabbit IFITMlike1

A

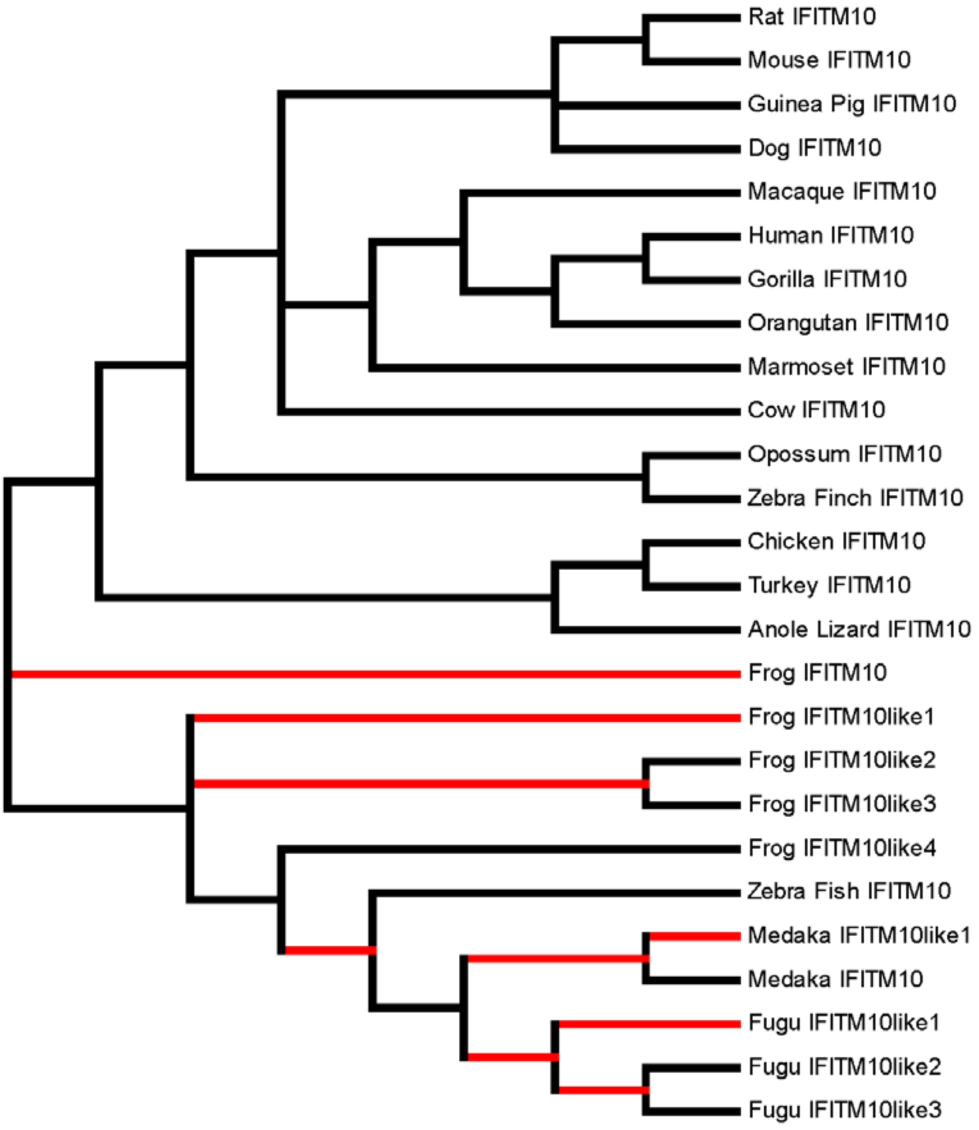

B

Figure S7. Positive selection analyses of IFITM genes with branch-site Rel model in DATAMONKEY. Red lines indicate branches under positive selection. A: positive selection in primate and rodent IR-IFITM dataset; B: positive selection in vertebrate IFITM10 dataset.
