# Supplementary material for: Evolutionary Dynamics of the Interferon-Induced Transmembrane Gene Family in Vertebrates
Source: PLoS One. 2012 Nov 15;7(11):e49265. doi: 10.1371/journal.pone.0049265 (PMC3499546; doi:10.1371/journal.pone.0049265)
Supplement: Table S2 — Gene conversion analyses of IR-IFITM genes. (DOC) [file pone.0049265.s009.doc]

| **Sequence1** | **Sequence2** | **Num Poly ^a^** | **Num**  **Dif ^b^** | **Tot Difs ^c^** | **BC KA**  **p-value ^d^** |
| --- | --- | --- | --- | --- | --- |
| **CDS** | | | | | |
| **Macaque IFITM-like6** | Macaque IFITM-like2 | 78 | 0 | 32 | 0.001 |
| **Macaque IFITM-like6** | Macaque IFITM-like1 | 69 | 0 | 43 | 0.001 |
| **Cow IFITM-like2** | Cow IFITM-like5 | 60 | 0 | 35 | 0.006 |
| **Cow IFITM-like1** | Cow IFITM-like5 | 58 | 0 | 36 | 0.006 |
| **Cow IFITM-like3** | Cow IFITM-like4 | 50 | 0 | 38 | 0.018 |
| **Dog IFITM-like6** | Dog IFITM-like4 | 141 | 0 | 44 | 0.001 |
| **Dog IFITM-like3** | Dog IFITM-like4 | 111 | 0 | 37 | 0.001 |
| **Dog IFITM-like3** | Dog IFITM-like6 | 81 | 0 | 44 | 0.001 |
| **Horse IFITM-like1** | Horse IFITM-like3 | 51 | 0 | 26 | 0.010 |
| **Horse IFITM-like1** | Horse IFITM-like2 | 50 | 0 | 26 | 0.014 |
| **Elephant IFITM-like1** | Elephant IFITM-like3 | 36 | 0 | 19 | 0.017 |
| **Intron** | | | | | |
| **Macaque IFITM-like6** | Macaque IFITM-like2 | 71 | 0 | 312 | 0.001 |
| **Macaque IFITM-like6** | Macaque IFITM-like1 | 42 | 0 | 325 | 0.001 |
| **Cow IFITM-like2** | Cow IFITM-like5 | 24 | 0 | 274 | 0.001 |
| **Cow IFITM-like1** | Cow IFITM-like5 | 20 | 0 | 266 | 0.001 |
| **Cow IFITM-like3** | Cow IFITM-like4 | 30 | 0 | 238 | 0.013 |
| **Dog IFITM-like6** | Dog IFITM-like4 | 20 | 0 | 2029 | 0.001 |
| **Dog IFITM-like3** | Dog IFITM-like4 | 28 | 0 | 1098 | 0.001 |
| **Dog IFITM-like3** | Dog IFITM-like6 | 32 | 0 | 2020 | 0.002 |
| **Horse IFITM-like1** | Horse IFITM-like3 | 32 | 0 | 387 | 0.001 |
| **Horse IFITM-like1** | Horse IFITM-like2 | 23 | 0 | 374 | 0.001 |
| **Elephant IFITM-like1** | Elephant IFITM-like3 | 60 | 0 | 638 | 0.001 |

**Table S2. Gene conversion analyses of IR-IFITM genes**

a, the recombinant amino acid sites between two sequences; b, the difference between the recombinant regions; c, total different amino acids between two sequences; d, the BC KA p-value <0.05 means [significant](app:ds:significant) difference between two sequences.
