# Supplementary material for: Evolutionary Dynamics of the Interferon-Induced Transmembrane Gene Family in Vertebrates
Source: PLoS One. 2012 Nov 15;7(11):e49265. doi: 10.1371/journal.pone.0049265 (PMC3499546; doi:10.1371/journal.pone.0049265)
Supplement: Table S3 — Twelve pairwise comparisons showing significantly higher dN than dS for IR-IFITM genes. (DOC) [file pone.0049265.s010.doc]

**Table S3. Twelve pairwise comparisons showing significantly higher dN than dS for IR-IFITM genes**

|  | **Gene** | **Gene** | **dN/dS** | **P-value** |
| --- | --- | --- | --- | --- |
|  | Rat IFITM-like2 | Rat IFITM-like3 | 3.978 | 0.013 |
|  | Human IFITM-like1 | Chimpanzee IFITM-like1 | 2.169 | 0.022 |
|  | Mouse IFITM-likeac | Mouse IFITM1 | 7.125 | 0.022 |
|  | Mouse IFITM-like1 | Mouse IFITM2 | 5.670 | 0.022 |
|  | Gorilla IFITM-likeac | Macaque IFITM-like5 | 1.815 | 0.033 |
|  | Cow IFITM-like4 | Cow IFITM-like5 | 1.749 | 0.041 |
|  | Gorilla IFITM3 | Chimpanzee IFITM-likeac | 1.731 | 0.044 |
|  | Human IFITM3 | Chimpanzee IFITM-likeac | 1.268 | 0.044 |
|  | Gorilla IFITM2 | Chimpanzee IFITM-likeac | 1.310 | 0.046 |
|  | Chimpanzee IFITM-likeac | Chimpanzee IFITM2 | 1.750 | 0.046 |
|  | Gorilla IFITM-like1 | Chimpanzee IFITM-likeac | 1.423 | 0.046 |
|  | Chimpanzee IFITM-likeac | Chimpanzee IFITM-like2 | 1.598 | 0.046 |
