# Supplementary material for: Evolutionary Dynamics of the Interferon-Induced Transmembrane Gene Family in Vertebrates
Source: PLoS One. 2012 Nov 15;7(11):e49265. doi: 10.1371/journal.pone.0049265 (PMC3499546; doi:10.1371/journal.pone.0049265)
Supplement: Table S4 — Positively selected sites in IR-IFITM genes detected using MEME method implemented in DATAMONKEY. (DOC) [file pone.0049265.s011.doc]

**Table S4. Positively selected sites in IR-IFITM genes detected using MEME method implemented in DATAMONKEY**

This summary table reports the distribution of synonymous (α) and non-synonymous (β) substitution rates over sites inferred by the MEME model, where the proportion of branches with β>α is significantly greater than 0. p-value is derived using a mixture of χ2 distributions, and q-values are obtained using [Simes' procedure](http://en.wikipedia.org/wiki/False_discovery_rate#Independent_tests), which controls the false discovery rate under the strict neutral null (likely to be conservative).

|  | **Site** | **ɑ** | **β(β=β^-^)** | **p-value** | **q-value** |
| --- | --- | --- | --- | --- | --- |
| Primate | 51 | 0.800 | 0.973 | 0.031 | 0.649 |
|  | 56 | 0.292 | 0.758 | 0.057 | 0.476 |
|  | 70 | 0.306 | 0.876 | 0.007 | 0.519 |
|  | 99 | 0.183 | 0.937 | 0.092 | 0.556 |
|  | 133 | 0.348 | 0.967 | 0.039 | 0.575 |
| Rodent | 37 | 0.473 | 0.717 | 0.082 | 0.695 |
|  | 70 | 0 | 0.599 | 0.088 | 0.912 |
|  | 125 | 0 | 0.935 | 0.081 | 0.586 |
|  | 130 | 1.428 | 5.843 | 0.058 | 1 |
| Marmoset | 12 | 0 | 0.510 | 0.034 | 1 |
|  | 16 | 0 | 6e-09 | 0.002 | 0.247 |
|  | 47 | 0 | 0.090 | 0.097 | 1 |
|  | 49 | 0 | 0.766 | 0.070 | 1 |
|  | 71 | 0 | 0.829 | 0.087 | 1 |
|  | 126 | 0 | 0.709 | 0.008 | 0.519 |
|  | 133 | 0 | 0.975 | 0.039 | 1 |
| frog | 112 | 0 | 7e-9 | 0.078 | 1 |
