# Supplementary material for: Evolutionary Dynamics of the Interferon-Induced Transmembrane Gene Family in Vertebrates
Source: PLoS One. 2012 Nov 15;7(11):e49265. doi: 10.1371/journal.pone.0049265 (PMC3499546; doi:10.1371/journal.pone.0049265)
Supplement: Figure S2 — Sequence alignment of the vertebrate IFITM5 genes. Alignment was used to reconstruct the Bayesian tree in Figure 2B. (PDF) [file pone.0049265.s002.pdf]

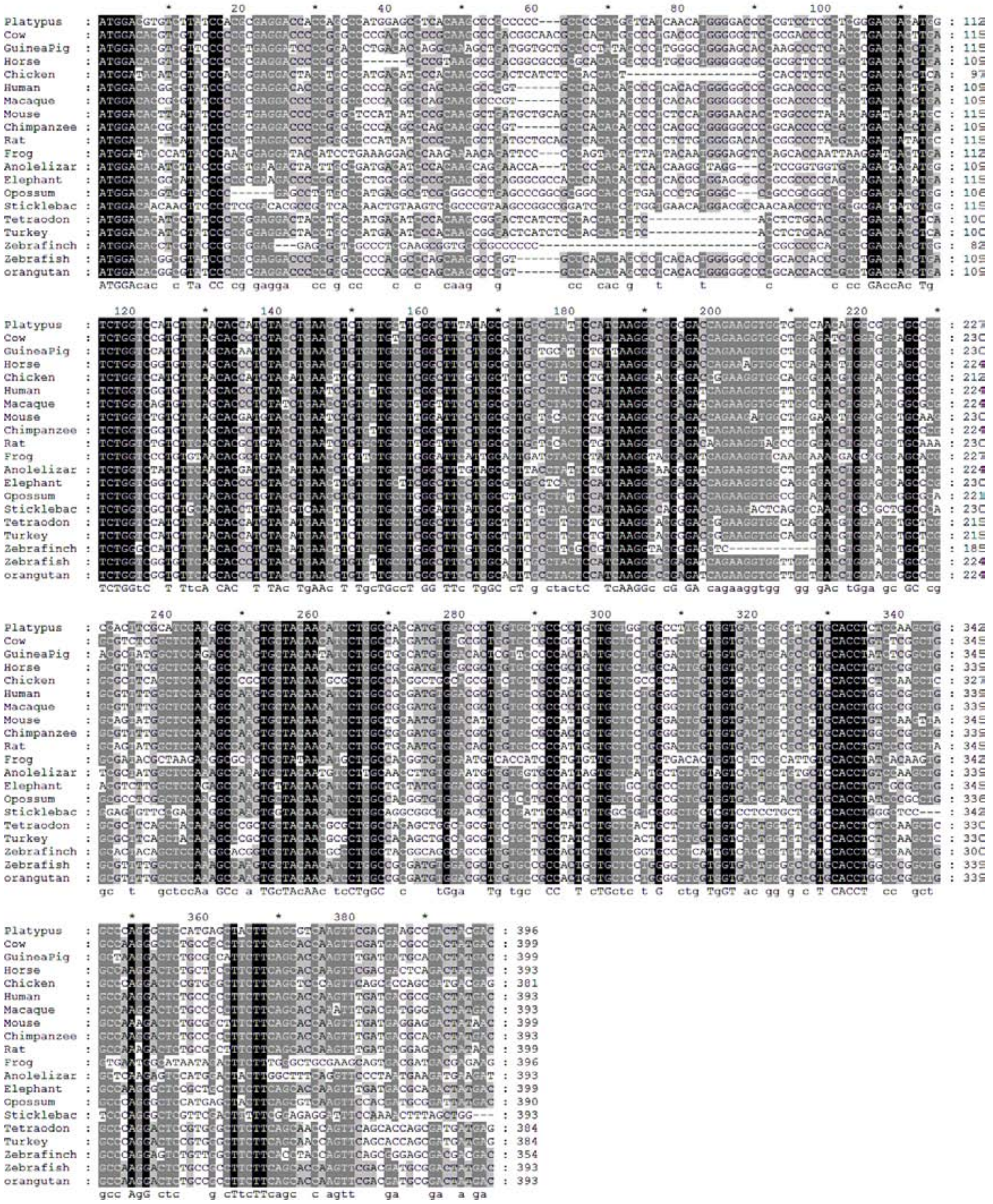

Figure S2 Sequence alignment of the vertebrate IFITM5 genes. This alignment was used to reconstruct the Bayesian tree in Figure 2B.
